# Can Complete Blood Count Parameters Predict Retinopathy of Prematurity?

**DOI:** 10.4274/tjo.galenos.2019.45313

**Published:** 2020-04-29

**Authors:** Ayşe İpek Akyüz Ünsal, Özge Key, Duygu Güler, İmran Kurt Omurlu, Ayşe Anık, Buket Demirci, Sema Dündar

**Affiliations:** 1 Aydın Adnan Menderes University Faculty of Medicine, Department of Ophthalmology, Aydın, Turkey; 2 Aydın Adnan Menderes University Faculty of Medicine, Department of Biostatistics, Aydın, Turkey; 3 Aydın Adnan Menderes University Faculty of Medicine, Department of Pediatrics Neonatology Division, Aydın, Turkey; 4 Aydın Adnan Menderes University Faculty of Medicine, Department of Medical Pharmacology, Aydın, Turkey

**Keywords:** Retinopathy of prematurity, complete blood count, risk prediction

## Abstract

**Objectives:**

To predict the risk of retinopathy of prematurity (ROP) development according to routine complete blood count (CBC) parameters.

**Materials and Methods:**

The medical records and CBC results of 150 premature neonates were retrospectively evaluated. As ROP develops 1 month after birth, first month CBC profiles of neonates without ROP (non-ROP), with ROP (ROP group), and those with Type 1, Type 2, and Stage 1+2 ROP were compared. Besides known statistical methods like Student’s t test, logistic regression and classification & regression tree (C&RT) analysis were also done to identify a reliable quantitative predictive parameter.

**Results:**

Mean gestational age and birth weight of the ROP group (n=99) and non-ROP (n=43) group were 29.39±3.43 and 32.05±2.20 weeks and 1382.44±545.30 and 1691.51±360.84 grams, respectively (p<0.001, p<0.001). Average hemoglobin (Hb) (p<0.001), hematocrit (HCT) (p<0.001), erythrocyte (p=0.005), mean corpuscular hemoglobin (MCH) (p=0.020), and MCH concentration (p=0.019) values of the ROP group were lower than those of the non-ROP group. Leukocyte was higher in the ROP group (p=0.018). Hb [odds ratio (OR)=0.668, 95% confidence interval (CI)=0.555-0.804, p<0.001], red cell distribution width (RDW) (OR=1.282, 95% CI=1.012-1.624, p=0.040), leukocyte (OR=1.157, 95% CI=1.053-1.271, p=0.002), and platelet (OR=0.997, 95% CI: 0.994-0.999, p=0.036) values differed significantly between the two groups. Platelet, MCV, and MCH parameters were found to be lower in the Type 1 ROP group compared to the Stage 1+2 ROP group (p<0.005). MCH was the most prominent predictor (cut-off: 34.43 pg) according to the results of C&RT analysis.

**Conclusion:**

As Hb plays an important role in oxygen transport, low levels of Hb and especially MCH may cause increased vascular endothelial growth factor secretion from the hypoxic retina, thereby causing ROP. Therefore, the results of this study are encouraging regarding the use of the abovementioned CBC parameters as a simple screening test to predict ROP.

## Introduction

Retinopathy of prematurity (ROP) is a retinal vascular disorder causing visual impairments like strabismus, amblyopia, cataract, glaucoma, and finally blindness.^1^ In order to prevent blindness related to ROP, simple, reliable, and predictive data are needed to distinguish infants at risk. This would reduce the number of cases with advanced ROP and enable timely treatment. In addition, unnecessary and risky ROP examinations in busy clinics may be avoided and the waste of time and effort prevented. As the proportion of surviving premature infants increases and the number of infants having the opportunity to be examined for ROP in the rural areas of developing countries decreases, the need for data to predict ROP is inevitable.^2,3^

Considering the pathophysiology of ROP, the hyperoxia and hypoxia phases influence not only angiogenic and inflammatory cytokine production in the retina but also the production of blood cells, their volume, and the production of cytokines in the bone marrow. Previous studies have investigated neutrophil-to-lymphocyte ratio (NLR), mean platelet volume (MPV), and thrombocyte, lymphocyte, nucleated and absolute nucleated red blood cell (RBC) counts.^4,5,6,7,8^

In this study, we analyzed complete blood count (CBC) profiles to identify a simple and prominent predictor for ROP, instead of investigating the parameters individually. Unlike the previous literature, we evaluated the same parameters 4 weeks after birth. The main aim of this study was to find a potential hematologic predictor at the time that ROP findings appear in the retina.

## Materials and Methods

This study was conducted in the ophthalmology and neonatology departments of a tertiary referral university hospital between May 2013 and 2016 and included 150 infants. Ethical approval was obtained from the local ethics committee (2016/885). The same ophthalmologist (A.İ.A.Ü.) examined the infants according to the standards of the International Committee for the Classification of ROP (ICROP).^9^ Initial screening was done at gestational age (GA) of 31 weeks for those born before 27 weeks and at 4 weeks after birth for those born after 27 weeks’ gestation. Although ROP screening for preterm infants born at <32 weeks GA and <1500 g birth weight (BW) is the common approach, the Neonatal Study Group in Turkey suggests evaluation of infants born at >32 weeks GA and >1500 g BW if the infant has a history of cardiopulmonary support and is at risk for developing ROP according to a neonatologist.^3^ Based on this reference, infants born at <36 weeks GA and <2670 g BW were also included in this study. Examinations were performed 1 hour after the instillation of 1% phenylephrine and 0.5% tropicamide. Funduscopic examinations were done by using binocular indirect ophthalmoscope, +28 diopter lens, pediatric speculum, and scleral depressor. Follow-up examinations and treatments were conducted according to the ICROP and ETROP criteria.^9,10^

The data of neonates born between 24 and 36 weeks GA with BW less than 2670 g (570-2670 g) were analyzed retrospectively. The infants were divided into 2 main groups: those with no signs of ROP were included in the “non-ROP” group, while infants with stage 1, 2, and 3 ROP were included in the “ROP group”. None of the infants developed stage 4, 5, or aggressive posterior ROP (APROP). All cases of type 1 ROP were treated with argon laser photocoagulation according to the ETROP criteria; none was treated with anti-vascular endothelial growth factor injection.

In this study, type 1 ROP infants (n=12) were also analyzed separately as a subgroup. The data of 12 infants with type 1 ROP were compared with those of non-ROP infants born at ≤32 weeks GA as a control-subgroup (n=24) and the stage 1+2 ROP subgroup. Furthermore, the same analyses were performed between the type 1 ROP and type 2 ROP groups (n=21).

Complete blood count results from 4 weeks after birth were obtained from the electronic database of our hospital. Hematocrit (Hct), hemoglobin (Hb), mean corpuscular volume (MCV), mean corpuscular hemoglobin (MCH), MCH concentration (MCHC), red cell distribution width (RDW), procalcitonin (PCT), mean platelet volume (MPV), platelet distribution width (PDW), red blood cell (RBC), white blood cell (WBC), neutrophil, lymphocyte, monocyte, eosinophil, basophil, and platelet counts, and neutrophil/lymphocyte ratio (NLR) were compared between the ROP and non-ROP groups. Blood groups were also analyzed for the prediction of ROP. Four infants who had culture-proven septicemia and 4 other infants who were transfused with blood products in the fourth week after birth were excluded from the study.

### Statistical Analysis

The normality of numeric variables was assessed using Kolmogorov-Smirnov test. Comparisons of normally distributed numeric variables between the groups were made by independent-samples t test. Descriptive statistics of the normally distributed numeric variables were presented as mean ± standard deviation. Mann-Whitney U test was used to compare non-normally distributed numeric variables between the groups and their descriptive statistics were presented as median (25^th^-75^th^ percentiles). Factors significantly associated with ROP were determined with binary logistic regression analysis. P values below 0.05 were considered statistically significant.

A recursive partitioning method called Classification and Regression Tree (C&RT) analysis was used both for regression and classification. Beginning with the entire data set, C&RT was constructed by splitting subsets of the data set using all predictor variables to create two child nodes repeatedly. The best predictor was chosen using a variety of impurity or diversity measures. The aim was to produce subsets of the data which are as homogeneous as possible with respect to the target variable.^11^ In our study, we used C&RT method in order to choose the best predictor and its cut-off point for ROP development.

## Results

One hundred and forty-two infants met the inclusion criteria of this study. The mean GA at birth of the whole study group was 30.20±3.34 weeks (range: 24-36 weeks) and the mean BW was 1476.04±515.53 g (range: 570-2670 g). The non-ROP group consisted of 43 infants and the ROP group had 99 infants. Mean GA at birth of the infants in ROP group was 29.39±3.43 weeks (range: 24-36 weeks) and the mean BW was 1382.44±545.30 g (range: 570-2670 g). These figures in the non-ROP group were 32.05±2.20 weeks (28-36 weeks) and 1691.51±360.84 g (780-2380 g), respectively (p<0.001, p<0.001). There was no correlation between blood group and ROP development (p=0.414).

**Figure 1:**
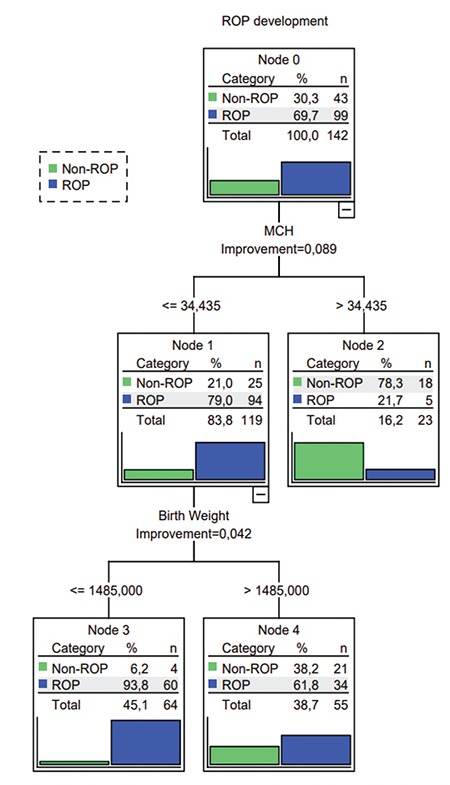
C&RT analyses of GA, birth weight, and counter blood cell parameters GA: Gestational age at birth, ROP: Retinopathy of prematurity

Hematologic parameters such as Hb, Hct, RBC, MCH, and MCHC values were lower and WBC count was higher in the ROP group. The results of the comparison of independent groups were summarized in Table 1. According to the results of logistic regression analyses of hematologic parameters obtained at postnatal 4 weeks, risk of ROP development was negatively correlated with Hb (odds ratio [OR]=0.668, 95% confidence interval [CI]: 0.555-0.804, p<0.001) and platelet count (OR=0.997, 95% CI=0.994-0.999, p=0.036) and positively correlated with RDW (OR=1.282, 95% CI=1.012-1.624, p=0.040) and WBC count (OR=1.157, 95% CI=1.053-1.271, p=0.002). When all parameters were analyzed with the C&RT method, the discrimination rate for ROP infants was 72.5%, that for non-ROP infants was 27.5%, and the accuracy of the model was 83.1%. The overall data of this analysis are shown in Figures 1 and 2. The most striking hematologic parameter was MCH and its cut-off value was 34.43 pg. MCH was a stronger predictor, even better than traditional risk factors such as GA at birth and BW. The accuracy of this prediction was 83.8%. BW was the second decisive factor with a cut-off point of 1485 g. The third value for prediction of ROP was WBC count, which had 78.2% accuracy at a cut-off point of 6325 mcL.

The descriptive analysis of subgroups including type 1 ROP, stage 1+2 ROP, type 2 ROP, and control-subgroup for GA and BW were as follows: 25.91±2.81 weeks, 28.52±2.28 weeks, 27.38±1.96 weeks, and 30.45±1.44 weeks and 973.75±462.14 g, 1248.92±429.60 g, 1077.24±350.56 g, and 1541.45±318.16 g, respectively. Comparison of type 1 ROP and type 2 ROP subgroups according to GA and BW was not statistically significant (p=0.246, p=0.062). Type 1 ROP and control-subgroup comparison revealed significantly lower Hb, Hct, RDW, and platelet values in the type 1 ROP subgroup (p<0.05) (Table 1). RDW value differed significantly between the type 2 ROP group and the control-subgroup (p=0.025). The comparison of type 1 ROP and stage 1+2 ROP subgroups showed that MCV, MCH, and platelet values were significantly lower in the type 1 ROP group, as shown in Table 2 (p<0.05).

## Discussion

In this study, the C&RT method was preferred to analyze the data in order to find the most predictive hematologic parameter and its cut-off point among premature infants at risk for ROP. After logistic regression analysis we identified the most important risk factor, but could not determine a cut-off point for risk prediction. In an attempt to find a cut-off point for risk prediction, we had to perform C&RT analysis. The main purpose of this study was to identify valuable predictive parameters with cut-off values to enable ROP risk assessment in daily practice. The analyses were done in two parts, the first consisting of only hematologic parameters with each other and the second including the most well-known risk factors like GA and BW. Both analyses converged on the same predictive parameter and cut-off point. If MCH was less than or equal to 34.43 pg at postnatal 4 weeks, the likelihood of developing ROP was 79%. MCH was found to be the most prominent predictive value among all parameters. As MCH represents the mean Hb value in red blood cells and Hb is indispensable for the distribution and presentation of oxygen into the tissues, this result is not surprising. At the end of the hyperoxic phase, infants with low MCH cannot respond to the increased need for oxygen in the developing retina and the second hypoxic phase begins with increased VEGF levels. The possible underlying mechanism of the MCH and ROP relationship could originate from the nitric oxide (NO) pathways. The relaxation of small resistance arteries is mostly achieved by NO, which must be maintained in a delicate balance in the vasculature.^12^ Hb in RBCs is not only responsible for oxygen transport and delivery, but also scavenges NO and produces Hb-NO complex.^12^ If excessive production occurs at the inflammation site, NO leads to vasodilatation, capillary leakage, and edema. NO reacts with superoxide and forms highly toxic peroxynitrite (ONOO^-^).^13^ It has been proven that peroxynitrite upregulates angiogenic factors such as VEGF, basic fibroblast growth factor (bFGF), and HIF-1alpha in human corneal limbal epithelial and human umbilical vein endothelial cell culture.^14^ It is clear that NO at the site of inflammation must be balanced by physiologic mechanisms, and this also explains the importance of having sufficient Hb in RBCs to prevent ROP.

To our knowledge, no previous study has described an association between MCH and ROP, but there are some studies that demonstrated significance of red cell parameters in ROP, partially supporting our study. Anemia was found to be an independent risk factor of ROP in Chinese infants born from multiple gestations.^15^ The declining trend in MCV and significantly increased RDW in the present study also support that the RBC/reticulocyte balance is worth investigating further to explain ROP pathophysiology. Indeed, Lubetsky et al.^7^ and Niranjan et al.^8^ demonstrated that increased nucleated RBC count on the first day of life is associated with intrauterine hypoxia and can be used for the prediction of ROP. Contrary to our study, no correlation was found between Hb levels and ROP according to the multiple logistic regression analyses in a study by Banerje et al.^16^ However, that study has a major methodological difference compared to ours, as they measured Hb levels of the premature infants on the first day of life.

According to logistic regression analyses, WBC count was significantly increased in our study. This increment was not due to any infection, as we excluded culture-proven septic premature infants at the beginning of our study by determining procalcitonin level (1.47 and 1.44 for non-ROP and ROP, respectively), which is used for prediction of infection. Although there was no sign of infection, lymphocyte, neutrophil, monocyte, and eosinophil counts tended to be higher in the ROP group and WBC count was significantly increased in our analyses, probably due to the ongoing inflammation of prematurity-related pathologies such as bronchopulmonary dysplasia, ROP, etc. Ashki et al.^14^ reported that macrophage, monocyte, and WBC infiltration causes NO release from tissues and NO consequently transforms into peroxynitrite. This highly toxic molecule increases angiogenic factors such as VEGF, b-FGF, and HIF1alpha. We assume that a similar underlying mechanism may also be valid for the ROP inflammation site as well. Similar to our study, Kurtul et al.^4^ studied the relationship between NLR and the development of ROP. They stated that inflammation in ROP can cause WBC and neutrophil counts to increase and they investigated NLR as a possible predictor for ROP in the first 24 hours of life. However, their results did not show NLR to be an independent predictor for ROP. In our study, a similar result was obtained for NLR at postnatal 4 weeks.

In this study, platelets were also evaluated for the prediction of ROP. Platelets store and carry bFGF, platelet-derived growth factor (PDGF), epidermal growth factor (EGF), matrix metalloproteinase (MMPs), and VEGF; alterations in volume and count could have a role in the pathophysiology of ROP. This is probably because they simultaneously contain both pro and anti-angiogenic factors.^17,18^ Clinical reports offer varying results regarding the clinical picture of ROP. Tao et al.^5^ stated that increased MPV was a marker of advanced stage ROP and attributed this to the larger platelets being more active than smaller ones in terms of carrying and storing VEGF. In our study, comparison of volume (MPV) and platelet counts between the ROP and non-ROP groups was statistically insignificant according to t tests. On the other hand, logistic regression analyses of the same parameters revealed that high platelet count was associated with low ROP risk (OR=0.997, 95% CI=0.994-0.999, p=0.036) and C&RT analyses supported the same result with a cut-off point of 442,500/µL. Premature infants with a platelet count under 442,500/µL had a 92.2% risk for ROP development. Similar to our study, Jensen et al.^6^ reported that thrombocytopenia is a risk factor for advanced stage ROP. A study by Yau et al.^15^ revealed that low platelet count is also an important risk factor for ROP in babies of multiple gestation.

Although patient numbers in the subgroups were insufficient to apply C&RT statistics, lower levels of Hb, Hct, RDW, MCV, MCH, and platelets in the type 1 ROP group in comparison to stage 1+2 ROP and control-subgroups (p<0.005) suggest the importance of oxygen transport by RBCs. This finding is worth considering because if there is not enough Hb and MCH to transport oxygen, it would not be as important as previously believed whether the given oxygen concentration is high or low, although oxygen concentration is one of the major risk factors for ROP.

No statistically significant difference was found between the gender of babies and ROP development. Furthermore, to our knowledge, the relationship between blood groups and ROP development has not been previously reported. Our study data did not reveal any significant correlation between blood groups and ROP development.

Unfortunately, further investigation of functional capacity is needed for all of the aforementioned cell types, as neonatal and preterm physiology completely differs from that of adults. Hematological parameters show extreme fluctuations in a developing premature infant.^19^ Keeping this in mind, our study design focused on the specific time period of postnatal 4 weeks to avoid variations in CBC profiles. As the aim of our study was to evaluate CBC in accordance with the retinal findings of premature infants and to find a predictive value, we preferred to assess the potential pathophysiologic effects of blood cells on the retina and retinal vascularization in a certain time period. Despite the retrospective nature of our study, the validity and significance of our clinical research is worth considering, because low Hb levels cause hypoxia in all tissues and in the retina as well, leading neonatologists to transfuse blood, which is an accepted risk factor for ROP. Prospective studies may provide more information regarding prediction, prevention, and treatment strategies for ROP.

## Conclusion

In conclusion, previous studies investigated hematologic parameters for prediction of ROP individually, but these parameters could be interrelated. In order to find the most important risk factor and a cut-off point to predict ROP, we evaluated all the hematologic parameters at the same time with a different statistical analysis method (C&RT). Even when taking the most well-known risk factors like GA and BW into account, C&RT analyses revealed that red cell parameters, especially MCH, was the most prominent risk factor with a cut-off point at 34.43 pg and should be carefully monitored in clinics. CBC profile screening may be an easy to perform, economic, and widely available test to predict ROP.

## Figures and Tables

**Table 1:**
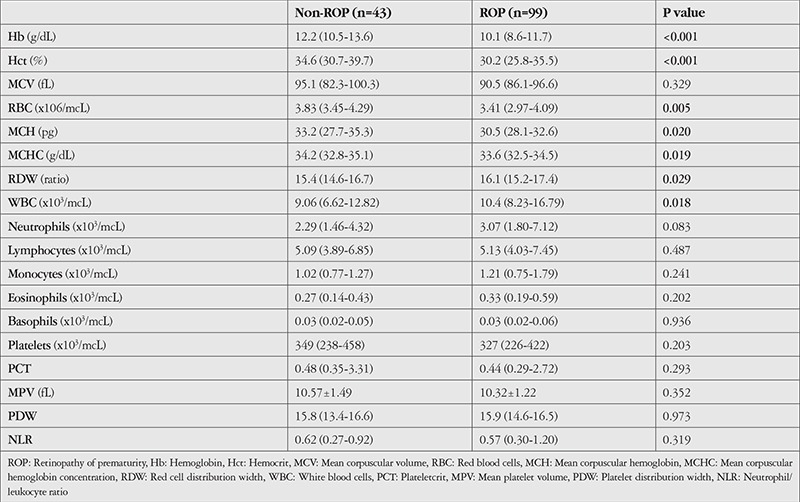
Mean hematologic parameters of the ROP and non-ROP groups at postnatal 4 weeks and p values for between-group comparisons

**Table 2:**
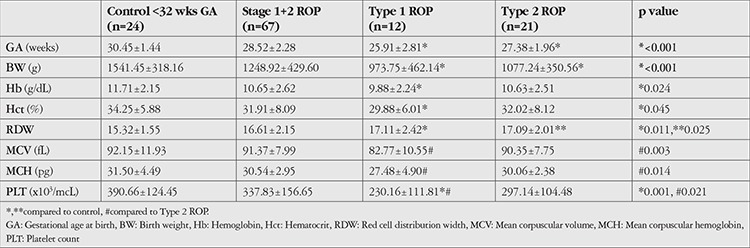
The comparison of groups born at <32 weeks GA

**Figure 2:**
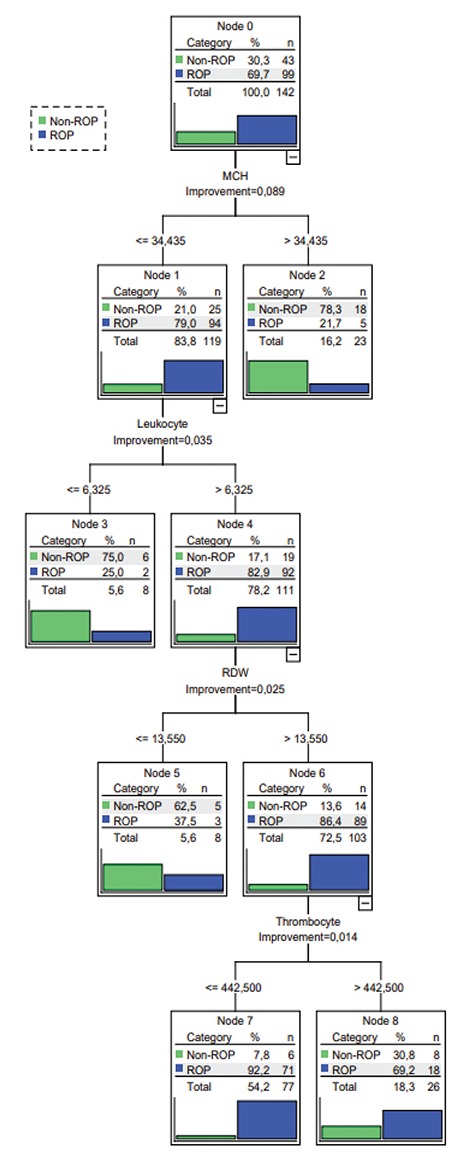
2. C&RT analyses for ROP only with counter blood cell parameters ROP: Retinopathy of prematurity
